# Characteristics and Outcomes of Patients Receiving Physical Therapy for Low Back Pain with a Nociplastic Pain Presentation: A Secondary Analysis

**DOI:** 10.1155/2023/5326261

**Published:** 2023-03-10

**Authors:** Abigail T. Wilson, Joseph L. Riley, Mark D. Bishop, Jason M. Beneciuk, Yenisel Cruz-Almeida, Joel E. Bialosky

**Affiliations:** ^1^University of Central Florida, School of Kinesiology and Rehabilitation Sciences, College of Health Professions and Sciences, Orlando, FL, USA; ^2^Musculoskeletal Research Lab, Institute of Exercise Physiology and Rehabilitation Science, University of Central Florida, Orlando, FL, USA; ^3^University of Florida, Department of Community Dentistry and Behavioral Science, Gainesville, FL, USA; ^4^Pain Research & Intervention Center of Excellence, University of Florida, Gainesville, FL, USA; ^5^University of Florida Department of Physical Therapy, Gainesville, FL, USA; ^6^Clinical Research Center, Brooks Rehabilitation, Jacksonville, FL, USA

## Abstract

**Introduction:**

Individuals with low back pain (LBP) may be classified based on mechanistic descriptors, such as a nociplastic pain presentation (NPP). The purpose of this secondary analysis was to examine the frequency and characteristics of patients with a NPP referred to physical therapy with LBP. Additionally, we characterized patients with LBP meeting the criteria for NPP by demographic, clinical, psychological, and pain sensitivity variables. Finally, we examined short- and long-term clinical outcomes in patients with a NPP compared to those without a NPP.

**Materials and Methods:**

Patients referred to physical therapy for LBP completed the Patient Self-report Survey for the Assessment of Fibromyalgia. Participants were categorized as “LBP with NPP” or “LBP without NPP” based on the threshold established in this measure. A rank sum test examined for differences in pain-related psychological factors and pressure-pain threshold between groups. Next, a Friedman test examined if LBP intensity and disability trajectories differed by groups at one and six months after initiation of physical therapy.

**Results:**

22.2% of patients referred to physical therapy for LBP met the criteria for a NPP. Patients with a NPP reported significantly greater disability, pain catastrophizing, depression, anxiety, and somatization compared to individuals without a NPP (*p* < 0.05). Pressure-pain threshold did not differ between groups (*p* > 0.05). Individuals with LBP with a NPP demonstrated nonsignificant, small to medium reductions in pain and disability at one and six months. Individuals experiencing LBP without a NPP demonstrated significant reductions in pain and disability in the short- and long term.

**Conclusion:**

Patients with LBP with a NPP displayed greater negative pain-related psychological factors but similar pain sensitivity compared to LBP without NPP.

## 1. Introduction

Low back pain (LBP) is the most common reason why patients seek rehabilitation in the world [1]. While rehabilitation is a recommended treatment approach in the management of LBP, [2–5] only small to moderate effect sizes for changes in LBP ratings and function are observed in experimental trials of interventions [6–9]. Large interindividual variability in response to treatments for pain may contribute to small average effect sizes and changes in clinical outcomes from these studies. Heterogeneity is observed within LBP and differences are often greater between individuals than between conditions [10]. This variability between patients has led to efforts to subgroup individuals based on clinical, somatosensory, or psychological characteristics [10–12]. Forming homogenous subgroups within a heterogeneous condition, such as LBP, is clinically relevant and has been proposed as a method of predicting clinical trajectories [13, 14].

One method of subgrouping patients with pain is based on mechanistic descriptors [15]. Pain is mechanistically classified as nociceptive (activation of nociceptors), neuropathic (lesion to the somatosensory system), and/or nociplastic (altered nociception without clear evidence of tissue damage) [15]. The term nociplastic was introduced in 2016 by the International Association for the Study of Pain to reflect the concept that neuroplastic changes occurring in chronic pain may progress to the point of pain transitioning from a symptom to a disease [16]. Musculoskeletal pain conditions, such as nonspecific LBP, may be classified as nociplastic pain [17]. Musculoskeletal pain conditions often have characteristics of each mechanistic classification [18] and subgroups of patients with LBP may have a nociplastic pain presentation (NPP) [18–23]. Given that nociplastic pain is an overarching classification encompassing a broad range of conditions, results from more studied nociplastic pain conditions (e.g. fibromyalgia [24]) may be applied to other conditions with subgroups of patients with a nociplastic pain presentation (NPP) (e.g. LBP) [25].

Fibromyalgia is a chronic pain condition of unknown etiology characterized by widespread pain with fatigue and sleep disturbances [26–28]. While a diagnosis in its own right, fibromyalgia is further recognized as an underlying contributor to some other chronic pain conditions [29] presenting along a polysymptomatic continuum termed “fibromyalgianess” [30]. About one-quarter of individuals with chronic LBP also report symptoms in multiple domains (widespread pain, sleep disturbances, etc.) consistent with the polysymptomatic presentation of fibromyalgia, [31, 32] suggesting a similar underlying nociplastic mechanism may be in effect in some people with cLBP. An overlapping, or mixed, presentation may occur in individuals with chronic LBP in which features of nociceptive and nociplastic pain are both present. The etiology of a NPP is multifactorial with the diverse patient presentation; however, limited research has directly compared the characteristics of patients with and without a NPP in LBP.

Therefore, the first purpose of this secondary analysis is to determine the percentage of a sample of patients referred to physical therapy with LBP who meet the criteria for fibromyalgia, and therefore a NPP, based on the Patient Self-Report Survey for the Assessment of Fibromyalgia. The second purpose is to characterize patients with LBP meeting the criteria for fibromyalgia by demographic, clinical, psychological, and pain sensitivity variables. The third purpose is to determine if short- and long-term pain and disability outcomes differ between individuals with LBP with and without a NPP. This secondary analysis is clinically relevant as it applies a measure of NPP, identifies biopsychosocial differences between patients experiencing LBP with and without a NPP, and examines clinical outcomes.

## 2. Methods

This is a secondary analysis of an observational, prospective cohort study conducted between December 2020 and August 2021. Data were collected from the following outpatient physical therapy clinics: six clinics within Brooks Rehabilitation in Jacksonville, Florida, USA, two clinics within the University of Florida Health in Gainesville, Florida, and one clinic within the University of Florida Health in Jacksonville, Florida. Thirteen physical therapists underwent IRB-01 training and a thirty-minute training session by the study coordinator to standardize data collection and recruitment methods. The University of Florida Institutional Review Board for Human Subjects Research approved this study and all participants provided written informed consent to enroll in the study.

### 2.1. Participants

Participants who were between 18 and 75 years old and receiving outpatient physical therapy for LBP were eligible to participate in the study. LBP was defined as pain between the inferior posterior margin of the ribs and the horizontal gluteal fold [33]. Participants who did not speak English, had a systemic medical condition known to affect sensation, or underwent a low back surgery or recovery from a fracture within the past six months were not eligible to participate in the study.

### 2.2. Study Overview

Participants referred to physical therapy for LBP underwent a standard evaluation at the discretion of the physical therapist. During the initial evaluation or first follow-up appointment, the patient's physical therapist informed potential participants of the research using an IRB-approved script and study flyer. Interested participants were screened for eligibility criteria by study staff. Eligible participants who consented to participate completed self-report demographic and clinical measures, the Patient Self-Report Survey for the Assessment of Fibromyalgia, psychological measures, and clinical outcomes upon enrollment. Clinical outcomes of LBP intensity and disability were collected at baseline, one month, and six months after study enrollment allowing for short- and long-term assessments. Study enrollment was within two weeks of the physical therapy evaluation. In addition to self-report measures, physical therapists measured pressure-pain threshold (PPT) at the low back and upper trapezius allowing for assessment of local and remote pain sensitivity. Self-report questionnaires were collected and managed using Research Electronic Data Capture (REDCap) tools hosted at the University of Florida. REDCap is a secure, online software designed for collecting data in research studies. Participants underwent physical therapy treatment at the discretion of the physical therapist for the duration of the study and interventions were not standardized.

### 2.3. Self-Report Demographic and Clinical Measures

Participants completed a self-report demographic form with age, sex, ethnicity, and race. The following clinical features of LBP were collected: duration of LBP in weeks, number of previous episodes, LBP intensity, and LBP-related disability.

#### 2.3.1. Pain Intensity

Participants rated their current, worst, and best LBP within the past twenty-four hours. These pain ratings were averaged, and the average LBP rating was reported and used in all analyses. LBP intensity was rated using a 101-point numerical pain rating scale (NPRS) where 0 = no pain and 100 = worst pain imaginable. A higher average pain rating indicates a higher LBP intensity. The NPRS demonstrates excellent psychometric properties [34–36]. The NPRS was completed at baseline, one month, and six months.

#### 2.3.2. Low Back Pain-Related Disability

LBP-related disability was measured with the Oswestry Disability Index (ODI), a reliable and valid measure of disability in patients with LBP [37]. A higher score on the ODI indicates a higher perceived LBP-related disability. The ODI was completed at baseline, one month, and six months.

#### 2.3.3. Patient Self-Report Survey for the Assessment of Fibromyalgia

The Patient Self-Report Survey for the Assessment of Fibromyalgia is a five-item self-report questionnaire that measures the severity of fibromyalgia symptoms [27, 28]. Participants check the number of locations of their pain using a body diagram and answer questions related to the associated symptoms of fibromyalgia, including the severity of fatigue, trouble thinking, feeling unrefreshed, abdominal pain, depression, and headaches [27]. Participants may receive up to a maximum score of thirty-one points with a higher score indicating greater severity of symptoms. Participants completed the questionnaire at baseline.

Prior research has reported a score of thirteen or higher on this measure indicates fibromyalgia [27]. The Patient Self-Report Survey for the Assessment of Fibromyalgia is sensitive and specific for diagnosing fibromyalgia. A score greater than thirteen was selected as this demonstrates the greatest sensitivity = 93.1% and specificity = 91.7% for diagnosing fibromyalgia against a gold standard diagnosis made by a rheumatologist [38]. The agreement between physicians and patient self-report classification is good for diagnosing fibromyalgia (83.4%, *k* = 0.67), providing support for this assessment's validity [39].

The Patient Self-Report Survey for the Assessment of Fibromyalgia is a screening tool intended for determining the prevalence of fibromyalgia in research. However, the questionnaire has been applied as a surrogate method to screen for subgroups of individuals with a nociplastic pain presentation in other populations, including myofascial pain [40].

### 2.4. Psychological Measures

#### 2.4.1. Symptom-Checklist-90-Revised (SCL-90R)

The SCL-90R is a ninety-item questionnaire that provides an overview of psychological symptoms in nine dimensions, including somatization, anxiety, and depression [41, 42]. Only the somatization, anxiety, and depression domains were included in this analysis. A higher score indicates greater psychological distress in that domain. The SCL-90R has been previously applied to patients with LBP [43]. Participants completed the SCL-90R at baseline.

#### 2.4.2. Pain Catastrophizing Scale (PCS)

The PCS is a thirteen-item questionnaire in which higher scores indicate higher pain catastrophizing. This measure may be applied to individuals with painful conditions and demonstrates good internal consistency, reliability, and validity [44–46]. Participants completed the PCS at baseline.

#### 2.4.3. Fear-Avoidance Beliefs Questionnaire (FABQ)

The FABQ includes a work and physical activity subscale with a higher score indicating greater fear-avoidance belief. The FABQ is a common measure in studies of individuals with LBP and is both reliable and valid [47–49]. Participants completed the FABQ at baseline.

#### 2.4.4. Tampa Scale of Kinesiophobia (TSK-11)

The TSK is a self-report measure of fear of movement that demonstrates acceptable psychometric properties [50]. Higher TSK scores indicate greater fear of movement due to pain. Participants completed the TSK-11 at baseline.

#### 2.4.5. Pain Self-Efficacy Questionnaire (PSEQ)

The PSEQ measures pain-related self-efficacy beliefs with higher scores indicating elevated levels of pain-related self-efficacy [51]. Participants completed the PSEQ at baseline.

#### 2.4.6. Pittsburgh Sleep Quality Index (PSQI)

The PSQI is a self-report measure assessing sleep quality and disturbances in the past month [52]. A cut-off score of five distinguishes good sleepers from poor sleepers with a sensitivity = 89.6% and a specificity = 86.5%. Adequate reliability and validity are observed in individuals with other chronic health conditions [53, 54]. Participants completed the PSQI at baseline.

### 2.5. Pain Sensitivity

#### 2.5.1. Pressure-Pain Threshold (PPT)

Physical therapists were trained by the study coordinator on the site of the PPT application, using the algometer, and applying pressure at a constant rate. A digital pressure algometer (Wagner Instruments FPX 25, Greenwich, CT) with a 1 cm diameter rubber tip was applied at 1 kgf/s ipsilateral to the same side of the patient's LBP medial to the posterior superior iliac spine and upper trapezius. The algometer was applied to a site local to the participant's LBP and remote as a behavioral measure of local and widespread changes in pain sensitivity. Widespread reductions in pain threshold are observed in individuals with fibromyalgia compared to healthy controls, suggesting an overall lessening of pain sensitivity [55]. Participants were instructed to indicate when the sensation first changed from pressure to pain (pain threshold). Participants then rated the pain at the threshold using the 101-point NPRS. This procedure was repeated two times and the average PPT was analyzed. PPT has good to excellent interrater reliability and test-retest reliability in patients with LBP [56].

### 2.6. Statistical Analysis

SPSS v. 27 (IBM, Armonk, NY) was used for all data analysis. Individuals who scored a thirteen or higher on the Patient Self-report Survey for the Assessment of Fibromyalgia met the criteria for fibromyalgia and, therefore, were categorized as LBP with NPP. Individuals who scored less than thirteen did not meet the criteria for fibromyalgia and were, therefore, categorized as LBP without NPP. Therefore, two groups were formed (1) LBP with NPP and (2) LBP without NPP. Characteristics of the total sample, as well as each group, were determined using descriptive statistics and frequency analysis for continuous and categorical variables, respectively.

The first purpose of this secondary analysis was to determine the percentage of a sample of patients referred to physical therapy with LBP who met the criteria for fibromyalgia, and therefore a NPP, based on the Patient Self-Report Survey for the Assessment of Fibromyalgia. A frequency analysis was conducted to determine the percentage of patients within each group.

The second purpose was to characterize patients with LBP meeting the criteria for fibromyalgia by demographic, clinical, psychological, and pain sensitivity variables. Due to the small sample of individuals with NPP (*n* < 20), nonparametric tests were used for data analysis. Group differences in demographic, clinical, and psychological characteristics were determined with a rank sum test for continuous variables and Chi-Square Analysis for categorical variables. An alpha level of 0.05 was established. The following demographic factors were examined: age, sex, race, and ethnicity. The following clinical factors were examined: average LBP rating on the NPRS, total percentage on the ODI, pain duration, and the number of previous episodes of LBP. The following psychological and sleep characteristics were examined: (a) somatization, depression, and anxiety collected as part of the SCL-90R; (b) pain catastrophizing collected with PCS; (c) fear-avoidance beliefs collected with FABQ-PA, FABQ-W; (d) kinesiophobia collected with TSK; (e) self-efficacy collected with PSEQ; and (f) sleep quality with PSQI. To examine differences between groups by local and remote changes in pain sensitivity, a separate rank sum test with PPT applied to the low back or upper trapezius as the dependent variable and group (LBP with NPP or LBP without NPP) as the independent variable was conducted. Due to the difference in the number of participants in each group and less than twenty participants in one group, hedge's *g* was calculated as a measure of effect size with the following formula: (*x*1 − *x*2)/√ ((*n*1 − 1) × *s*1^2^ + (*n*2 − 1) × *s*2^2^)/(*n*1 + *n*2 − 2)). Effect sizes were interpreted as follows: 0.2 = small, 0.5 = medium, 0.8 = large. [57]

The third purpose was to determine if short- and long-term pain and disability outcomes differ between individuals with NPP and those without a NPP. A Friedman test with pairwise comparisons determined changes in clinical pain intensity at one month and six months while accounting for baseline scores for patients with LBP with NPP and patients with LBP without NPP. A Friedman test with pairwise comparisons was repeated to determine changes in the ODI at one month and six months while accounting for baseline scores for patients with LBP with a NPP and patients with LBP without a NPP.

## 3. Results

97 participants were screened for eligibility criteria and 61 enrolled in the study. 54 participants were analyzed with baseline measures and 50 completed all follow-up measures at one month (93% follow-up rate). 41 participants completed all follow-up measures at six months (76% follow-up rate). During the four weeks, participants underwent physical therapy at the clinician's discretion. Physical therapy treatment was not standardized.

### 3.1. Frequency of Patients Receiving Physical Therapy for LBP with NPP

22.2% (*n* = 12) of patients with LBP met the threshold for a diagnosis of fibromyalgia, and therefore a NPP, based on the Patient Self-Report Survey for the Assessment of Fibromyalgia at baseline.

### 3.2. Characterization of Patients with LBP and NPP by Demographic, Clinical, and Psychological Factors

#### 3.2.1. Demographic Factors

As demonstrated in Table 1, in general, the total sample was middle-aged (mean age = 51 years old), female (73.10%), and not Hispanic or Latino (95.10%). Our sample demonstrated racial diversity (57.50% Caucasian, 31.10% African American, 4.90% Asian, 1.60% American Indian, and 4.90% other). Age, sex, race, and ethnicity did not significantly differ between patients with LBP and NPP and patients with LBP without NPP (*p* > 0.05).

#### 3.2.2. Clinical Factors

Average LBP intensity and disability at baseline were moderate for the total sample (mean ± SD pain = 50.28 ± 22.00 and disability = 36.82 ± 18.60%). Average back pain duration and a number of previous episodes indicated the total sample was predominantly individuals with chronic LBP. [33] Pain duration (*p*=0.18) and previous number of episodes (*p*=0.18) did not differ by group. As shown in Table 1, people with NPP reported higher pain intensity on average than people without; however, the difference was not statistically significant (*p*=0.19). Patients with LBP and NPP reported significantly higher disability (*p*=0.01) [37]. Collectively, individuals with LBP and NPP display significantly higher levels of disability compared to those with LBP without NPP.

#### 3.2.3. Psychological and Sleep Factors

**Figure 1:**
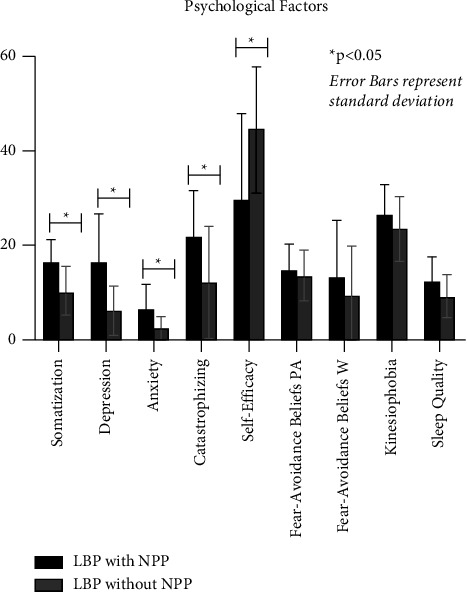
Pain-related psychological factors by NPP.

All negative pain-related psychological factors significantly differed between groups, with the exception of fear-avoidance beliefs. As demonstrated in Figure 1, somatization (*p* < 0.01, *g* = 1.18), depression (*p* < 0.01, *g* = 1.85), and anxiety (*p* < 0.01, *g* = 1.13) were significantly higher in patients with LBP with NPP compared to LBP without NPP. Pain catastrophizing was also significantly higher in patients with LPB with NPP compared to LBP without NPP (*p* < 0.01, *g* = 0.83). Measures of fear-avoidance beliefs were not significantly different between groups (FABQ-PA *p* = 0.67, *g* = 0.19, FABQ-W*p* = 0.32, *g* = 0.39 TSK *p* = 0.16, *g* = 0.43). Individuals with LBP with NPP also displayed significantly lower levels of pain-related self-efficacy (*p* = 0.01, *g* = 1.09). Sleep quality, while worse in people with NPP, did not significantly differ between groups (*p* = 0.06, *g* = 0.66) yet a medium to large effect size was observed. People with NPP had worse sleep quality than those without NPP although the difference did not quite reach the 5% threshold needed to reject the null hypothesis (*p* = 0.06).

#### 3.2.4. Pain Sensitivity

**Figure 2:**
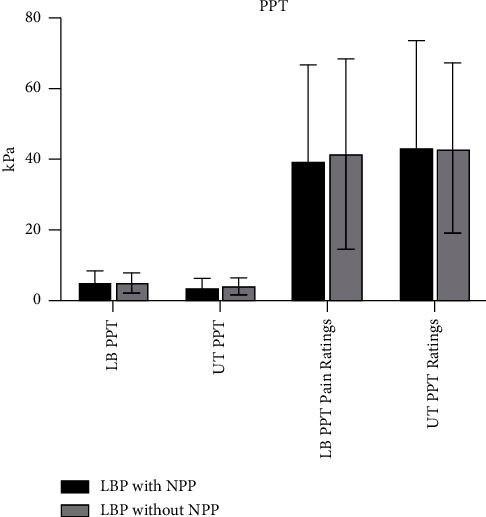
Pressure-pain threshold by NPP.

As demonstrated in Figure 2, PPT applied to the lower back and upper trapezius did not differ by the presence or absence of NPP (lower back *p*=0.89, *g* = 0.03; upper trapezius *p*=0.67, *g* = 0.21). Pain ratings at threshold also did not differ between groups (lower back *p*=0.82, *g* = 0.09, upper trapezius *p*=0.89, *g* = 0.01).

### 3.3. Short- and Long-Term Clinical Pain Intensity and Low Back Pain-Related Disability

**Figure 3:**
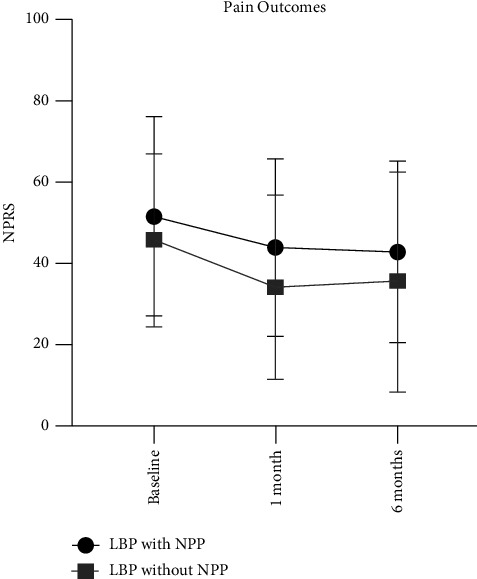
Short- and long-term pain outcomes after physical therapy.

As demonstrated in Figure 3, the pain did not significantly decrease (*p*=0.09) for patients with LBP with NPP between baseline to one month (*g* = 0.29) and six months (*g* = 0.36). Although not significant, effect sizes suggest small to medium reductions in pain in the short- and long-term pain for patients with LBP with NPP. For patients with LBP without NPP, pain significantly reduced (*p* < 0.01) between baseline to one month (*g* = 0.51) and six months (*g* = 0.51). Medium effect sizes for changes in pain were observed for patients with LBP without NPP. Effect sizes for differences in pain ratings between those with and without a NPP were small at baseline (*g* = 0.26), one month (*g* = 0.44), and six months (*g* = 0.27).

**Figure 4:**
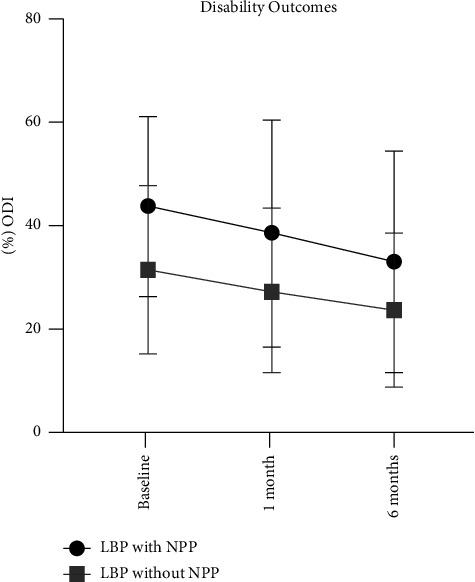
Short- and long-term disability outcomes after physical therapy.

As demonstrated in Figure 4, disability did not significantly change over time for patients with LBP with NPP (*p*=0.09) between baseline to one month (*g* = 0.27) and six months (*g* = 0.51). Although not significant, a small reduction was observed at one month and a medium reduction in disability was observed at six months. Disability significantly (*p*=0.02) reduced over time for patients displaying LBP without NPP between baseline to one month (*g* = 0.25) and six months (*g* = 0.51). Effect sizes for differences in disability between those with and without a NPP ranged from large to medium at baseline (*g* = 0.96), one month (*g* = 0.54), and six months (*g* = 0.40).

## 4. Discussion

Clinical features, such as pain duration, are part of the criteria for determining nociplastic pain in patients with musculoskeletal pain [23]. Furthermore, clinicians often categorize patients with NPP based on a patient's history of diffuse or widespread chronic pain that is easily elicited and disproportionately high to the nature of the injury [58]. In this study, we categorized each individual's pain presentation based on a self-report questionnaire for NPP and then examined group differences in psychosocial factors. Our main findings were that 22% of our patients experiencing LBP met the criteria for fibromyalgia and, therefore, likely have pain of nociplastic origin (NPP). Patients with LBP with NPP reported significantly higher pain catastrophizing, depression, anxiety, and somatization compared to patients without a NPP with a large effect size noted. However, groups were not differentiated by demographic, clinical, and pain sensitivity factors. This research is clinically relevant as our results also indicate that individuals with LBP without NPP demonstrated significant reductions with a small to medium effect size in pain and disability in both the short- and long-term. However, individuals with LBP with NPP displayed nonsignificant, small to medium reductions in pain and disability in the short- and long-term.

### 4.1. Patients with LBP Presenting with NPP

Approximately one quarter (22%) of our sample of patients with LBP met the threshold for a NPP based on the Patient Self-Report Survey for the Assessment of Fibromyalgia at baseline. Other investigators have reported higher frequencies of a NPP in patients with chronic LBP. A prior study reported 48% of patients with chronic LBP were identified as fibromyalgia positive based on the 2011 Fibromyalgia survey (Widespread Pain Index and Symptom Severity) [19]. In this study conducted in patients with chronic LBP, individuals who were positive for fibromyalgia also demonstrated significantly higher LBP intensity. However, our results indicate that the only clinical feature that differentiated patients with LBP and NPP was disability. Consistent with prior literature, [19] individuals with LBP plus NPP reported greater pain-related disability on the ODI compared to individuals with LBP without NPP. Although we anticipated pain duration and intensity may also differentiate the groups, these features were similar regardless of NPP. As a secondary analysis, we did not specifically recruit for the presence of NPP nor exclude participants based on the duration or intensity of symptoms. Differences in eligibility criteria may also contribute to the lack of difference in average pain ratings between patients.

### 4.2. Characteristics of Patients with LBP Presenting with NPP

The results of this study suggest that pain-related psychological factors may be a key differentiating characteristic in patients with and without a NPP. Higher pain catastrophizing, depression, anxiety, and somatization, as well as lower pain self-efficacy, were reported in patients with LBP with NPP compared to those without a NPP. Multiple pain-related psychological factors may impact the pain experience for individuals with NPP. Consistent with prior literature, catastrophizing is associated with higher pain perception in patients with fibromyalgia [55] and is positively associated with depression in patients with fibromyalgia [59, 60]. Depression is highly prevalent in individuals with fibromyalgia [61] and antidepressant medications may be prescribed as a treatment for this condition [62]. This study adds to this body of literature by also demonstrating higher somatization, higher anxiety, and lower pain-related self-efficacy are observed in individuals with LBP with NPP compared to those without a NPP. Higher somatization, depression, and anxiety levels are predictive of impaired disability in patients with fibromyalgia [63]. Collectively, elevated negative pain-related psychological factors contribute to the pain experience in patients with LBP with NPP and may represent therapeutic targets to explore in future clinical trials. These results are clinically relevant because these variables may represent novel pain-related psychological factors to screen and monitor in patients with LPB with NPP [25, 64–66]. However, this should be examined in future clinical trials.

In our sample, a clinically feasible measure of pain sensitivity (pressure-pain threshold) did not differentiate patients with LBP with NPP from patients without a NPP. This result is inconsistent with prior literature demonstrating patients with fibromyalgia display elevated thermal and pressure sensitivity compared to healthy controls [60, 67]. Patients with LBP and fibromyalgia have heightened pain sensitivity measured with pressure-pain threshold [19]. The term “central sensitization” is commonly used as an indicator of nervous system hyperexcitability and a broad explanation for a patient's widespread pain symptoms [68, 69]. While nociplastic pain and central sensitization are different constructs, [16, 23] our results are consistent with prior literature demonstrating the poor association between pain sensitivity measures and self-report assessments of central sensitization (Central Sensitization Index (CSI)). The CSI [70] is a valid and reliable questionnaire [71] to quantify the severity of central sensitization symptoms in patients with chronic pain [22]. The CSI is also not correlated with changes in pain sensitivity measured with quantitative sensory testing [72, 73].

### 4.3. Relationship between NPP Status and Clinical Outcomes

A NPP has important clinical implications as it also impacts short- and long-term reductions in pain and disability after physical therapy. Our results indicate individuals with LBP without NPP demonstrate significant reductions in pain and disability at one and six months with small to medium effect sizes. However, individuals with LBP with a NPP demonstrate small to medium but nonsignificant reductions in pain and disability at one and six months. This questionnaire has been previously applied as a surrogate measure of NPP to examine the prevalence of this presentation in musculoskeletal pain conditions, such as LBP [19, 74]. Consistent with the results of this study, patients with myofascial pain who displayed higher scores on this questionnaire, and therefore a greater NPP, demonstrated poorer clinical outcomes after physical therapy. Patients with NPP are a clinically important subgroup that reports higher levels of pain and disability [75] with smaller reductions in these outcomes after physical therapy treatment compared to nociceptive pain. We add to this body of literature by demonstrating the presence (or absence) of a NPP may be informative for short- and long-term pain and disability outcomes.

### 4.4. Strengths and Limitations

There are strengths and limitations worth considering when interpreting the results of this analysis. A strength of our study is we administered a multidimensional assessment of pain including multiple psychological questionnaires, pain sensitivity, and clinical outcomes. Additionally, our results are clinically relevant with the inclusion of short- and long-term outcomes. However, a limitation of our study is that the Patient Self-Report Survey for the Assessment of Fibromyalgia has not been previously examined for validity or reliability in individuals with LBP. Treatment was also provided at the discretion of the physical therapist which could influence clinical outcomes. Additionally, as a secondary analysis, we did not specifically enroll patients who met the criteria for a NPP. As a result, we had an unequal number of participants in each group with a small sample size of individuals with a NPP.

## 5. Conclusion

In conclusion, approximately one-quarter of patients referred to physical therapy for LBP were identified as having a presentation consistent with LBP and NPP. Patients with LBP plus NPP were further characterized by greater disability, negative pain-related psychological factors, and decreased short- and long-term reductions in pain intensity and disability. Pain catastrophizing, depression, anxiety, and somatization are psychological factors rehabilitation providers may choose to screen and monitor in individuals with a NPP. Individuals with LBP with a NPP also demonstrate nonsignificant decreases in pain and disability at one month and six months after rehabilitation compared to individuals without a NPP. These results are clinically meaningful as pain-related psychological factors may represent novel therapeutic targets.

## Figures and Tables

**Table 1:** Baseline demographic and clinical characteristics for the total sample and by the group.

	Total sample (*n* = 54)	LBP without NPP (*n* = 42)	LBP with NPP (*n* = 12)	*p* value
Demographic characteristics				
Age (years)	51.05 ± 17.02	52.78 ± 17.69	51.33 ± 15.48	0.49
Sex (% female)	73.10	66.70	83.30	0.27
Ethnicity (% Hispanic)	4.90	2.40	16.70	0.06
Race (%)				
Caucasian	57.50	61.9	66.70	0.21
African American	31.10	23.8	25.0	
Asian or Pacific Islander	4.90	4.80	8.30	
American Indian or Alaskan native	1.60	2.40	0.00	
Other	4.90	7.10	0.00	
Clinical characteristics				
Average LBP rating (101-point NPRS)	50.28 ± 22.00	46.18 ± 19.96	55.88 ± 23.13	0.19
ODI (%)	36.82 ± 18.60	31.41 ± 16.20	46.83 ± 16.06	0.01^*∗*^
Previous episodes of back pain (number)	55.89 ± 155.79	37.98 ± 76.43	147.16 ± 314.17	0.18
Duration (weeks)	135.51 ± 242.83	140.83 ± 292.06	141.42 ± 140.49	0.18

*Note*. *p* value represents group differences with ^*∗*^ indicating statistical significance *p*  <  0.05. Values represent mean ± SD.LBP = low back pain, NPRS = numerical pain rating scale, ODI = Oswestry Disability Index.

## Data Availability

The data set is available from the corresponding author (Abigail.Wilson@ucf.edu) upon request.
